# Stent Grafts Can Convert Unusable Upper Arm Arteriovenous Fistulas into a Functioning Hemodialysis Access: A Retrospective Case Series

**DOI:** 10.3389/fsurg.2017.00013

**Published:** 2017-02-27

**Authors:** Charudatta S. Bavare, Tiffany K. Street, Eric K. Peden, Mark G. Davies, Joseph J. Naoum

**Affiliations:** ^1^Department of Cardiovascular Surgery, Methodist DeBakey Heart & Vascular Center, Houston Methodist Hospital, Houston, TX, USA; ^2^Department of Surgery, Division of Vascular and Endovascular Surgery, Lebanese American University, Beirut, Lebanon

**Keywords:** hemodialysis access, arteriovenous fistula, stent graft, Viabahn, fistula stenosis, dialysis access salvage

## Abstract

**Introduction:**

Not all newly created arteriovenous fistulas (AVFs) successfully mature and develop into a functioning access for hemodialysis. Percutaneous transluminal angioplasty (PTA) and balloon-assisted maturation (BAM) have been utilized to either treat flow-limiting stenoses or to promote and accelerate maturation. We hypothesized that unusable upper arm AVFs can be rescued by conversion to a functional access using the percutaneous placement of a stent graft (SG).

**Methods:**

Clinical data on 12 patients with an early non-usable upper arm AVF underwent percutaneous revision using SGs. There were six brachial–cephalic, three brachial–basilic, and three brachial–brachial vein transposition AVFs.

**Results:**

All patients had either at least two or more stenoses (>2 cm) within the fistula conduit, or a long segment stenosis (>4 cm) in combination with shorter segment stenoses. Nine patients had failed PTA. Three patients had failed BAM at outside access centers. All patients were referred for failure to achieve access cannulation and concomitant hemodialysis through the AVF. SGs were placed retrograde toward the arterial anastomoses and ranged in diameter (6, 7, and 8 mm in four, seven, and one patients, respectively). The average length of the SG was 10 cm (range 5–15 cm). All SGs were post-balloon dilated at the time of placement. All AVFs were salvaged, and patients were able to maintain functional use of their access with cannulation occurring through the SG. The primary patency rate at 6 and 12 months was 91% [95% confidence interval (CI), 56–98%] and 65% (95% CI, 32–87%), respectively (*n* = 11 and 5 at risk, respectively). The secondary patency rate at 6 and 12 months was 100 and 72% (95% CI, 46–93%), respectively (*n* = 11 and 7 at risk, respectively).

**Conclusion:**

This report outlines a successful initial experience using SGs to rescue, preserve, and convert an unusable upper arm AVF into a functioning hemodialysis access.

## Introduction

A patent functioning hemodialysis access, either graft or fistula, is critical in ensuring effective treatment for patients with end-stage renal disease. The implementation of clinical practice guidelines of the Dialysis Outcome Quality Initiative (K/DOQI) (1) has lead to movement toward a fistula-first initiative and an overall reduction in mortality for patients on hemodialysis.

The literature on the outcome of the use of stent grafts (SGs) in salvaging failed arteriovenous (AV) accesses is limited. Several studies have reported the outcomes for the use of bare metal stents and SGs to treat venous stenoses that exhibit elastic recoil after balloon angioplasty (2–5). Shemesh and colleagues (6) published a randomized prospective study suggesting that the use of SGs may be superior to bare metal stents for the treatment of cephalic arch stenoses in autogenous AV accesses.

The purpose of this case series is to examine the outcome of attempted salvage of early non-usable (primary non-maturation) AV fistulae (AVF) with SGs.

## Materials and Methods

### Study Design

This was a retrospective review of 12 patients who underwent placement of an SG for non-usable AVF (primary non-maturation) over a 2-year period. The Viabahn^®^ Endoprosthesis (W.L. Gore and Associates, Inc., Flagstaff, AZ, USA) was utilized in all cases. Collected data included patient demographics and medical comorbidities, along with dimensions of the SG placed. Hospital and clinic records were reviewed, and patients contacted to obtain follow-up data as part of an Institutional Review Board-approved protocol.

### Study Setting

This study was performed in an academic medical center with 1,000 beds in a catchment area of 5 million people. During the study period, a total of 672 primary AVF creations were performed.

### Study Population

Twelve patients (58% women; mean age 65 ± 10 years, range 46–81 years) who presented with a non-usable and/or abandoned AVF. The demographics, comorbidities, and type of AVF are described in Table 1. Nine patients had failed percutaneous transluminal angioplasty (PTA) (*n* = 9) to correct documented stenosis. Three patients had balloon-assisted maturation (BAM) attempted at outside access centers and failed (Table 2). The indication for SG placement in this group was access preservation and use.

**Table 1:** **Patient demographics, comorbidities, and type of arteriovenous fistula (AVF) present**.

Patient characteristics	Number (%)
Women	7 (58)
Men	5 (42)
End-stage renal disease on dialysis	12 (100)
Congestive heart failure	3 (25)
Hypertension	11 (92)
Diabetes	11 (92)
Hyperlipidemia	4 (33)
Stroke	4 (33)
**Type of AVF**	
Brachial–cephalic	6 (50)
Brachial–basilic vein transposition	3 (25)
Brachial–brachial vein transposition	3 (25)

**Table 2:** **Interventions on the arteriovenous fistula access prior to stent graft placement**.

Interventions	Number (%)
Percutaneous transluminal angioplasty	9 (75)
Balloon-assisted maturation	3 (25)

### Technique

**Figure 1:**
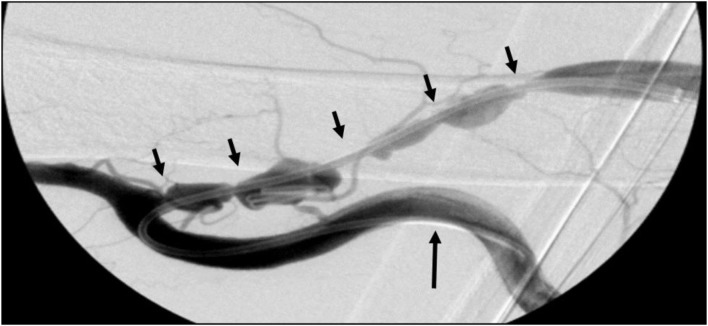
**Right brachial–basilic vein transposition fistulogram**. A 4-Fr glide catheter is shown advanced retrograde into the brachial artery (large arrow) to perform an arteriogram with concomitant imaging of the arteriovenous fistula and its multiple areas of stenosis (small arrows).

**Figure 2:**
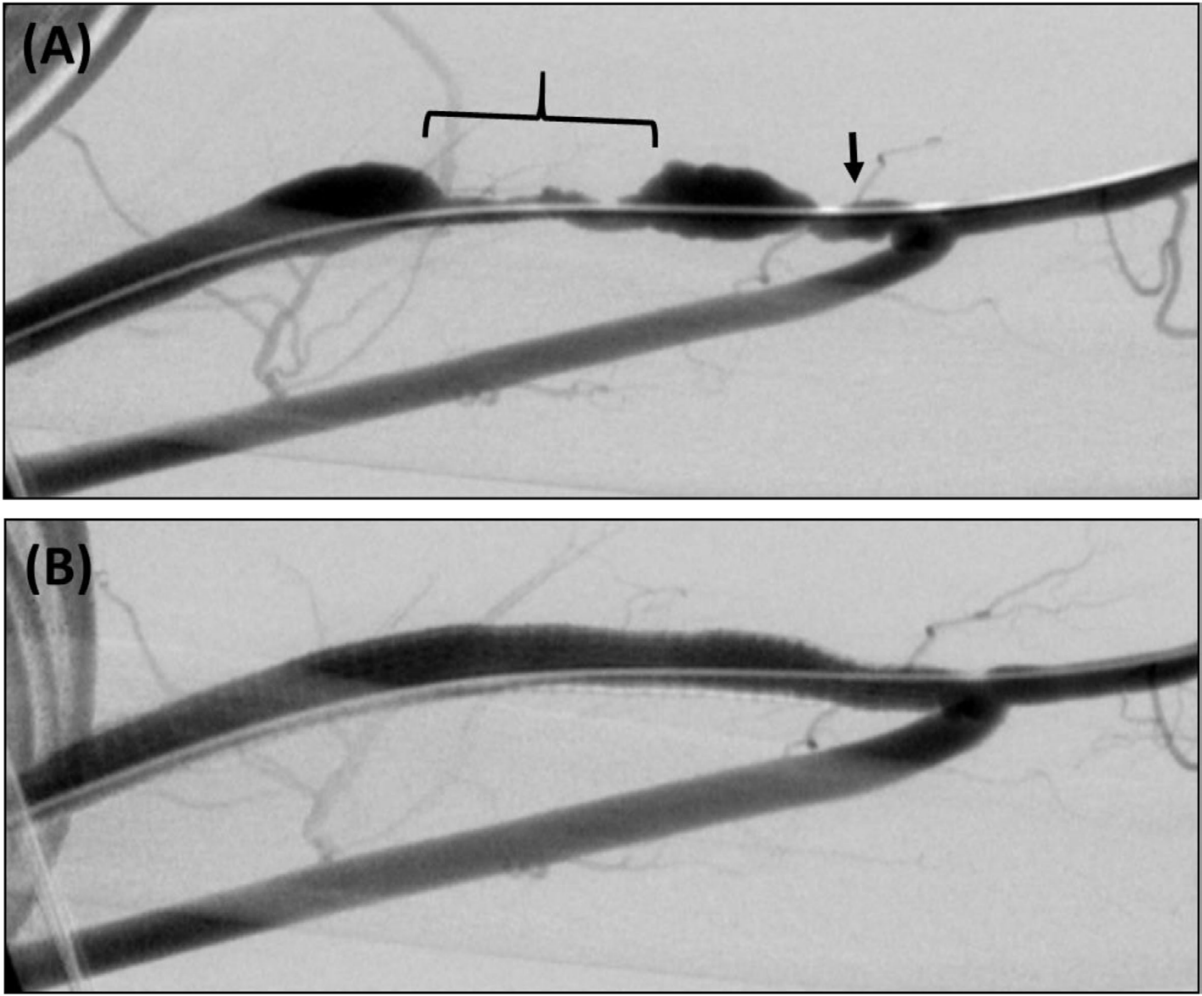
**(A)** Brachial–cephalic arteriovenous fistula with short-segment (arrow) and long-segment (bracket) areas of stenosis. **(B)** Retrograde fistulogram showing successful treatment with a 7 mm × 15 cm Viabahn SG.

A single retrograde ultrasound-guided puncture technique was utilized at the hemodialysis site to access the fistula. In general, a fistulogram was performed to delineate and characterize the areas of stenoses. Often, retrograde access into the brachial artery was required and achieved by manipulating a 0.035-mm angled glide wire (Terumo, Somerset, NJ, USA) from the venous stick into the brachial artery and then advancing a 4-Fr glide catheter (Glidecath^®^, Terumo) to perform an arteriogram with concomitant imaging of the AVF (Figure 1). This proved appreciably useful in instances where a juxta-anastomotic stenosis was present. Once the treatment area was identified, selection of the SG diameter was tailored to accommodate the diameter of the native fistula at the normal or stenosis-free segment. The Viabahn Endoprosthesis is a flexible, self-expanding nitinol stent lined with expanded polytetrafluoroethylene (ePTFE) that is approved to treat superficial femoral arterial occlusive disease. The SG was positioned and deployed with post-deployment angioplasty. Care was taken not to cross the SG into the arterial anastomosis in all cases. Technical success was routinely defined by completion fistulogram in Figure 2. Patients were typically discharged on the day of the procedure after clinical assessment and were assessed during routine follow-up with clinical success defined as successful dialysis access use. Typically the treated fistula was accessed within 2 weeks of the procedure. We postulate that this period of time allows for the inflammatory reaction generated by the SG to add to the venous and tissue wall thickness surrounding the SG.

Dialysis units were cleared to access the AVF through the segment of the vein containing the SG, thus directly puncturing through the graft. The duration of successful use of the access was determined from patient history and physical examination at the outpatient clinic visits. None of the patients had reports of incomplete or suboptimal dialysis treatments from the dialysis units. Duplex scans were not performed routinely when the AV access was functioning without clinical evidence of stenosis or malfunction.

### Definitions

Primary patency (PP) was defined as the time from SG placement until first access failure or intervention aimed at maintaining patency. Secondary patency (SP) was defined as the interval from SG placement to abandonment of fistula. Clinical success was defined as preservation of AV access after the outflow restoration with covered stent placement in the AVF. The patient’s status after discharge was followed through clinic records, and patients were seen in clinic and evaluated by physical exam. They were contacted to determine access complications and patency since their last visit at our institution. Survival was also confirmed by querying the Social Security Death Index.

### Statistical Analysis

Measured values are reported as percentages or means ± SD where appropriate. The patency rates were calculated by Kaplan–Meier analysis.

## Results

At the time of presentation, all patients were on hemodialysis through a tunneled dialysis catheter. All patients had a previously created but unusable AVF. The mean duration of the access was 3.6 months (range 2–4.5 months). Intraoperative venograms did not demonstrate central venous stenoses or arterial inflow disease in any of the patients. There were six brachial–cephalic upper arm, three brachial–basilic upper arm, and three brachial–brachial with vein transposition AVFs. The brachial–basilic and brachial–brachial AVF were two-stage procedures. All patients were referred for failure to achieve access cannulation and concomitant hemodialysis through the AVF. Patients had either at least two or more stenoses (>2 cm) within the fistula conduit, or a long segment stenosis (>4 cm) in combination with shorter segment stenoses. Nine patients had failed PTA. Three patients had failed BAM at outside access centers.

Stent graft sizes used were 6, 7, and 8 mm in diameter in four, seven, and one patients, respectively. The average length of the SG was 10 cm (range 5–15 cm) (Table 3). All SGs were post-balloon dilated at the time of placement. Technical success was achieved in all cases (100%); no residual stenosis was observed. There were eight AVF localized to the left upper arm and four to the right. Mean follow-up was 11 ± 7 months. There was no perioperative mortality. Seventy-eight percent of patients were alive at 1 year. The PP rate at 6 and 12 months was 91% [95% confidence interval (CI), 56–98%] and 65% (95% CI, 32–87%), respectively (*n* = 11 and 5 at risk, respectively). The SP rate at 6 and 12 months was 100 and 72% (95% CI, 46–93%), respectively (*n* = 11 and 7 at risk, respectively). None of the patients had access-related complications such as erosion or infection. One patient at long-term follow-up was found to have a pseudoaneurysm at the site of dialysis access puncture, which was repaired with placement of an SG.

**Table 3:** **Diameter and length measurements of the implanted stent grafts**.

Device characteristics	Number (%)
**Diameter (mm)**	
6	4 (33)
7	7 (59)
8	1 (8)
**Length (mm)**	
5	1 (8)
10	8 (67)
15	3 (25)

## Discussion

The “failing to mature” AVF can be defined as a surgically created AVF that failed to properly expand without stenosis and become usable for the purpose of hemodialysis within 8–12 weeks after its creation (7). This failure could clinically manifest as difficulty in cannulation, low flows, or both. The “failing to mature” AVF is caused by intrinsically poor native vessels or by post-surgical derangements. In this series, the AVF failed to mature exclusively due to venous stenosis within the AVF segment. The fistula-first initiative as per the KDOQI guidelines has led to creation of more native fistulas as primary access, in effect leading to the increase in incidence of primary failure rates ranging between 23 and 48% (8–14). Lower maturation rates may effectively reduce the functional patency of an AVF to a level approaching that of prosthetic AV grafts (15). Failure of maturation also means protracted hemodialysis through tunneled catheters, increasing the risk of infectious complications, and central venous stenosis (11).

Numerous techniques have been used to improve fistula function by addressing the cause of malfunction, including venous and arterial angioplasty, stent placement, thrombectomy, venous branch ligation, fistula superficialization, banding, interposition vein grafts, transposition, and extensive preoperative imaging (16–18). The technique of BAM needs special mention as a commonly used therapeutic technique to help facilitate fistula maturation, with reported success up to 85% (19). However, there is a lack of evidence describing the use of long-segment SGs to salvage unusable AVFs that have failed previous rescue attempts with PTA or BAM. All patients who underwent SG placement in our series had failed salvage treatment by the latter two techniques.

In the setting where a venous stenosis exhibits significant elastic recoil following angioplasty, the use of a stent is recommended to maintain luminal diameter and improve patency. In an early series by of eight patients with multiple AV graft failures, Naoum and colleagues (20) placed self-expanding nitinol stents for the treatment of a venous outflow stenosis and showed a 25% SP at 6 months. However, bare stents stimulate proliferation of intimal hyperplasia, leading to in-stent stenosis and eventual failure. The long-term results with the use of stents for the treatment of venous stenoses in AVF are similar to those obtained with angioplasty alone (21). SGs, however, have shown to have superior SP rates at 6 months compared to angioplasty alone in the setting of failing AVG (22).

Vesely and colleagues (23) retrospectively studied 51 patients who underwent angioplasty of a graft-related stenosis and required salvage with either a bare stent or SG. The PP of the vascular access was 81, 70, and 54% at 1, 3, and 6 months, respectively. The SP was 89, 82, and 74% at 3, 6, and 12 months, respectively. The authors concluded that bare stents and SG are useful in salvaging failed angioplasty procedures and maintaining patency of the hemodialysis graft. Furthermore, Viabahn SG has been shown to improve the patency of failing hemodialysis grafts with either venous outflow stenosis or occlusion. Stent-graft patency was 94.7 and 82.1% at 12 and 24 months, respectively. SGs improved freedom from reintervention rates and overall patency rates of failing AVGs (24). Similarly, in a retrospective review of 44 patients with failing AVGs, 11 were treated with PTA alone and 33 with the placement of a Viabahn SG. At 12 months, 87.8% of grafts treated with SG were functional compared to 36.4% of those with PTA alone (25). Cephalic arch stenosis is a cause of dysfunction in autogenous AVFs that often require multiple reinterventions with angioplasty. Shawyer and associates inserted six Viabahn SGs to treat residual stenosis after angioplasty and five for angioplasty induced rupture. Primary access patency rates were 81.8% at 6 months and 72.7% at 12 months (26). These reports demonstrate that SG can improve patency rates beyond those achieved with successful or for the treatment of failed PTA. More importantly, as in our series, SG can salvage the access and extend its use for hemodialysis.

Our use of SG in the setting of abandoned AVF for primary non-maturation is unique in its description and indication of use. We postulate that a 2-week maturation period of the access is needed to allow what we believe is the inflammatory response to generate thickening of the vessel wall and tissues in contact with the SG segment. We believe that this may contribute to decreased needle site bleeding following cannulation required during hemodialysis treatment. Our patency rates at 12 months compare favorably with the above cited reports.

Pseudoaneurysm formation at the site of access puncture is a foreseen complication associated with the use of ePTFE SG. Pseudoaneurysms are more commonly seen with ePTFE AVG for dialysis. Multiple reports describe repeated needle cannulation of dialysis grafts—particularly at the same location—leading to damage and breakdown of the graft material and subsequent pseudoaneurysm formation (27, 28). A systematic rotation of needle punctures to alternate sites may prove beneficial in this instance (29).

This retrospective case series reports the successful initial experience using SGs to rescue early unusable AVFs, converting them into a functioning hemodialysis access with no procedural related mortality or morbidity. With this revision, all access cannulation for hemodialysis occurs through the segment containing the SG. We propose a new name for this novel technique: a “Stentula” (SG and fistula).

**Figure 3:**
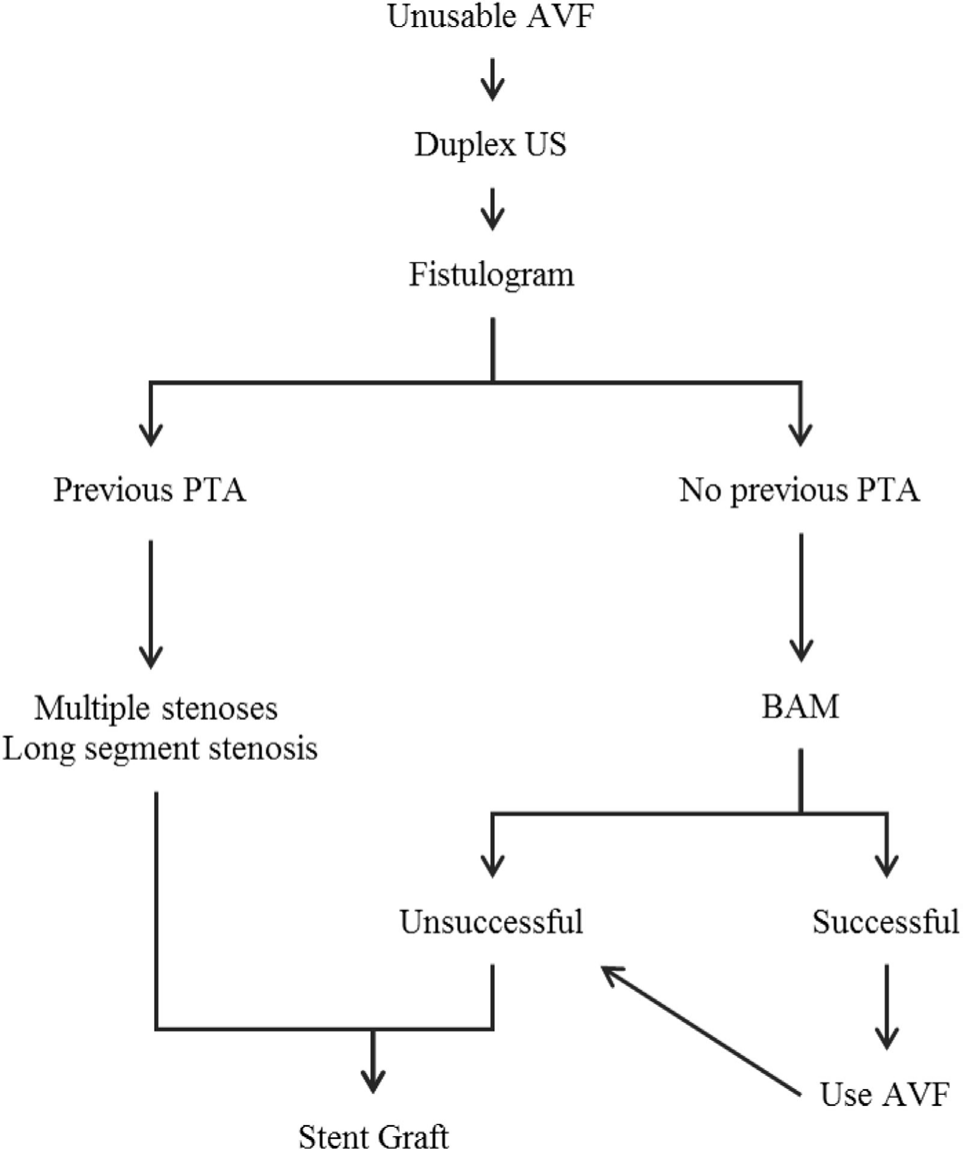
**Algorithm for salvage of unusable AVFs and conversion to a stentula**. AVF, arteriovenous fistula; US, ultrasound; PTA, percutaneous transluminal angioplasty; BAM, balloon-assisted maturation.

Limitations of this study include a small sample size, a retrospective analysis of the database, and a non-randomized study population. With such a small set of patients at the reported time points, the Kaplan–Meier estimates can be misleading and should be interpreted with caution. We realize that further work needs to be carried out to validate this initial early experience in this subset of dialysis patients. Based on this early experience, we propose an algorithm to guide the selection and treatment of patients for this procedure (Figure 3). This novel solution to treat failing or non-maturing abandoned AVFs cements the clinical utility of combining an SG with a fistula.

## Conclusion

Use of SGs to treat complications of dialysis access has been well documented. Our description and use in a failing to mature AVF plagued with stenoses is unique, safe, and yields patency rates that allow access salvage and its maintenance for hemodialysis. However, more studies and longitudinal follow-up are needed to validate the initial findings of this technique.

## Ethics Statement

Study data was obtained *via* a retrospective review of the patient’s medical records and standardized telephone interviews conducted with the patient as part of an IRB-approved protocol using preexisting data in a HIPAA compliant manner.

## Author Contributions

CB is the vascular fellow who contributed to manuscript writing and figure editing. TS contributed to patient follow-up and data gathering. EP is the advisor who contributed to manuscript review and writing. MD contributed to manuscript review, data set analysis, and editing. JN contributed to manuscript writing, review, operating surgeon, table creation, analysis.

## Conflict of Interest Statement

The authors declare that the research was conducted in the absence of any commercial or financial relationships that could be construed as a potential conflict of interest.
